# Association between blood microbiome and type 2 diabetes mellitus: A nested case‐control study

**DOI:** 10.1002/jcla.22842

**Published:** 2019-02-04

**Authors:** Jing Qiu, Hui Zhou, Yang Jing, Chen Dong

**Affiliations:** ^1^ Department of Epidemiology and Statistics, School of Public Health, Jiangsu Key Laboratory and Translational Medicine for Geriatric Disease Medical College of Soochow University Suzhou Jiangsu China; ^2^ Suzhou Industrial Park Centers for Disease Control and Prevention Suzhou China

**Keywords:** 16S ribosomal RNA, blood microbiome, nested case‐control study, type 2 diabetes mellitus

## Abstract

**Background:**

Although recent studies have indicated that gut microbiome dysbiosis was significantly associated with the onset of type 2 diabetes mellitus (T2DM), information on the role of blood microbiome in T2DM development is scarce.

**Methods:**

Fifty incident T2DM cases and 100 matched non‐T2DM controls were selected from a prospective cohort study of “135.” The composition of the blood microbiome was characterized using bacterial 16S ribosomal RNA (16S rRNA) gene sequencing from pre‐diagnostic blood sample. The amplicons were normalized, pooled, and sequenced on the Illumina MiSeq instrument using a MiSeq Reagent Kit PE300 v3 kit.

**Results:**

Totally, 3 000 391 and 6 244 227 high‐quality sequences were obtained from T2DM patients and non‐T2DM controls, respectively. The mean diversity of the blood microbiome (Simpson, Chao1 and Shannon indices) was not different between two groups at baseline. At genus level, the *Aquabacterium, Xanthomonas, *and *Pseudonocardia* were presented with lower abundance, while *Actinotalea, Alishewanella, Sediminibacterium,* and* Pseudoclavibacter *were presented with higher abundance among T2DM cases compared to those in non‐T2DM controls. As the results shown, participants carried the genus* Bacteroides* in blood were significantly associated with a decreased risk for T2DM development, with 74% vs 88% (adjusted OR: 0.367, 95% CI: 0.151‐0.894). However, participants carried the genus* Sediminibacterium* have an increased risk for T2DM, with adjusted OR (95% CI) being 14.098 (1.358, 146.330).

**Conclusions:**

Blood microbiome may play an etiology role in the development of T2DM. These findings would be useful to develop microbiota‐based strategies for T2DM prevention and control.

## 1 INTRODUCTION

Type 2 diabetes mellitus (T2DM) affects more than 440 million individuals worldwide. In China, the prevalence of T2DM has been reported to be 10.9%, with 35.7% of the population having abnormal glucose homeostasis.^1^, ^2^ In addition to the metabolic‐related factors, smoking, drinking, and inherited genetic factors are considered to be significantly associated with T2DM development. However, these factors only partially explain the pathogenesis of the disease.^1^, ^3^, ^4^, ^5^ Therefore, there is a need to improve the scientific knowledge about the causes of T2DM and to provide more guidance for the disease prevention and control.

“Balanced” gut microbiota is critical to maintain the host energy and metabolism equilibrium. Moreover, microbial dysbiosis in the human gut has been recognized as an important risk factor for the development of obesity‐related diseases, including hypertension and T2DM.^6^, ^7^, ^8^ During the past two decades, accumulative evidence has demonstrated that enteric bacterial products (eg lipopolysaccharides [LPS]) could cross the impaired intestinal barrier to reach peripheral circulation and contributed to the low‐grade chronic inflammatory state.^9^, ^10^ More recently, the results from several studies have suggested that both serum LPS and LPS‐binding protein levels were closely associated with obesity, metabolic syndromes, T2DM, and T2DM‐associated arterial stiffness,^11^, ^12^, ^13^ indicating that impaired intestinal barrier plays an etiology role in these chronic diseases development.

Blood in healthy humans is generally considered as a “sterile” environment. However, a number of sequence‐based and ultramicroscopic studies have reported that the gut microbiota could translocate into blood due to increased intestinal permeability.^14^, ^15^, ^16^, ^17^ In a recent study on a large general population, Amar et al^18^ observed that blood microbiota dysbiosis was significantly associated with the onset of cardiovascular events. Although more and more studies have suggested the presence of apparent associations between gut microbiome or enteric bacterial products and the onset of T2DM, there is a lack of information on the role of blood microbiome in T2DM development. Therefore, in the present study nested in a “135” cohort in the Chinese city of Soochow, we directly assessed the microbiome in blood by high‐throughput sequencing of the 16S ribosomal RNA (16S rRNA) gene using pre‐diagnostic blood samples collected from T2DM cases and controls and compared two groups for baseline overall blood microbiome composition and relative abundance of specific bacterial taxa.

## 2 METHODS

### 2.1 Study participants

The participants included in the present study were nested in a “135” cohort study, which is an ongoing prospective study and aims to investigate risk factors for chronic diseases including diabetes mellitus. Details on the methodology of the “135” study have been described before.^19^ In brief, total 5782 adults, aged from 35‐70 years, were recruited between March 2013 and September 2013 from Soochow, China. The demographic characteristics of each participant including weight, height, and blood pressure were collected by face‐to‐face interview. The first round follow‐up survey was conducted in 2015. T2DM cases were defined as incident cases (for those with no previous history of diabetes and had never used diabetic medication at the time of the baseline survey), and a diagnosis of T2DM was confirmed by a physician, or the reported use of T2DM medication at the time of the follow‐up survey.

In the present nested case‐control study, 100 controls were matched to 50 T2DM cases with sex and age (5 years). Pregnant women and individuals with cancers, chronic viral hepatitis, renal failure, chronic enteritis, and diarrhea were excluded. Additionally, no subject (in both T2DM and control groups) had taken antibiotic, probiotic, or prebiotic products since two months before the sample collection. This study received approval from the Ethics Committee of SIPCDC (Suzhou Industrial Park Centers for Disease Control and Prevention) in accordance with the 1975 Declaration of Helsinki. Informed consent was obtained from all individual participants.

### 2.2 Sample collection and laboratory measurement

Blood samples were taken by venipuncture after at least 8‐hour overnight fast. Tubes were centrifuged at 3000 *g* for 10 minutes at room temperature for separation. Plasma samples were frozen at −80°C for storage as quickly as possible. Fasting plasma glucose (FPG), triglyceride (TG), total cholesterol (TC), low‐density lipoprotein cholesterol (LDL), high‐density lipoprotein cholesterol (HDL), alanine aminotransferase (ALT), and aspartate aminotransferase (AST) were measured using an autoanalyzer (Olympus AU640, Tokyo, Japan).

### 2.3 DNA extraction, 16S rRNA gene amplification, and sequencing

Sample processing, DNA isolation, and PCR steps were conducted in a laminar air flow bench, illuminated with a UV lamp prior to use in order to avoid possible contaminants. Blood bacterial genome DNA was isolated from each plasma sample using Qiagen's QIAmp DNA kit (Qiagen Inc, Germantown, MD, USA). The V5‐V6 regions of 16S rRNA gene were PCR‐amplified using primers (forward primer, 5′‐CCTACGGGNGGCWGCAG; reverse primer, 5′‐GACTACHVGGGTATCTAATCC). Every reaction contained bacterial‐free blank control as negative controls for the quality confirmation. Moreover, each plate was sequenced on one MiSeq run with two duplicated quality controls and one negative control sample (lysis buffer and kit reagents only) included. The amplicons were normalized, pooled, and sequenced on the Illumina MiSeq instrument using a MiSeq Reagent Kit PE300 v3 kit (Illumina, CA, USA).

### 2.4 Derivation of microbiome data and quality controls

Sequence reads processing was performed using QIIME (Quantitative Insights Into Microbial Ecology, QIIME: http://qiime.org/) package v1.8. The join_paired_ends.py was used to stitch paired reads together by parameters of −*j* 45 −*p* 5 (≥45 bp overlapped and ≤5% unmatched bp between the paired reads). After that, sequencing reads were de‐multiplexed by removing barcode and primers. Low‐quality reads with phred score <30 were filtered out. Additionally, chimeric sequences were removed. The processed sequences were subjected to subsampled open‐reference operational taxonomic unit (OTU) at 97% sequence identity. The diversity and richness of the bacteria in the blood samples were calculated using several estimates, which consisted of the level of OTUs, Chao 1, Shannon, and Simpson indices.

### 2.5 Statistical analysis

Statistical analyses were performed using SAS 9.4 software (SAS Institute, Cary, NC, USA). Independent *t* tests and Mann‐Whitney *U* test were applied for continuous variables. For categorical variables between groups, we used either the Pearson chi‐square or Fisher's exact test. In order to analyze the associations between bacteria taxa and T2DM risk, subjects were categorized into two groups as carriers and non‐carriers of the pathogens because of low relative abundance of most pathogens in blood. The non‐carrier meant that the individual did not have sequence read for the specific pathogen. Logistic regression was used to estimate odds ratios (OR) and 95% confidence intervals (CIs) for measuring the association of specific blood pathogen with T2DM risk. Potential confounder factors including BMI, blood pressure, smoking, drinking, TC, and TG were adjusted. In the present study, we limited our analysis of bacterial phyla to those with mean relative abundance ≥0.01%. For lower level taxa (class to genus), we limited analysis to those with mean relative abundance ≥0.0001%. All tests of significance were two‐sided, and *P* < 0.05 was considered statistically significant.

## 3 RESULTS

### 3.1 Characteristics of the studied population

Demographic characteristics of cases (participants who developed T2DM) and controls (participants who did not develop T2DM) are shown in Table 1. T2DM cases and non‐T2DM controls in the present study were similar with respect to the matching factors of age and gender. In addition, smoking, drinking, baseline FPG did not significantly differ between cases and controls. However, participants who developed T2DM were more likely to be with hypertension, increased levels of TC, TG, and LDL at baseline. As expected, the plasma level of HDL in control group was apparently lower than that in T2DM group at baseline.

**Table 1:** Baseline characteristics of study population

Variables	T2DM (n = 50)	Control (n = 100)	*P*
Age (years)	51.64 ± 6.18	51.98 ± 8.05	0.775
Males (%)	35 (70.00)	64 (64.00)	0.465
Smoking (%)	21 (42.00)	45 (45.00)	0.727
Drinking (%)	9 (18.00)	12 (12.00)	0.318
Hypertension (%)	12 (24.00)	6 (6.00)	0.001
FPG (mmol/L)	6.12 (5.95, 6.43)	6.05 (5.23, 6.28)	0.453
BMI (kg/m^2^)	25.14 ± 2.88	23.42 ± 2.93	0.001
WC (cm)	87.11 ± 6.81	82.83 ± 8.17	0.001
SBP (mmHg)	128.64 ± 20.61	119.68 ± 14.50	0.007
DBP (mmHg)	82.78 ± 12.94	77.96 ± 9.82	0.023
ALT (U/L)	25.00 (17.00, 41.00)	18.00 (13.00, 25.00)	0.001
AST (U/L)	23.00 (18.00, 29.00)	21.00 (19.00, 27.00)	0.324
TC (mmol/L)	4.92 ± 0.97	4.54 ± 0.87	0.017
TG (mmol/L)	2.04 (1.34, 3.09)	1.26 (0.93, 1.79)	0.000
HDL (mmol/L)	0.99 (0.84, 1.35)	1.17 (0.95, 1.48)	0.013
LDL (mmol/L)	3.16 (2.49, 3.60)	2.80 (2.32, 3.60)	0.057

Numerical data were expressed as mean ± SD or median (inter‐quartile range). Categorical data were expressed as percentage.

### 3.2 Sequencing data summarization

The total number of reads obtained from 150 participants was 9 968 238. After filtering and removing the chimeric sequences, we obtained 3 000 391 high‐quality sequences from the participants developed T2DM (60 007/sample) and 6 244 227 sequences from non‐T2DM controls (62 442/sample), respectively. As the results shown in Table 2, there were 453 OTUs detected, including 330 OTUs and 403 OTUs in cases and controls, respectively. These OTUs could be classified into 10 phyla and 248 genera. Among of them, the phylum *Proteobacteria* predominated, representing approximately 99.59% of the bacteria. The other two common phyla were* Bacteroidetes* and *Firmicutes*, with average relative abundances of 0.15% and 0.13%, respectively (Table S1).

**Table 2:** Sequencing data of blood microbes in T2DM and control groups

Groups	Raw sequences	Superior sequences	OTU number	Numbers of different classification orders				
				Phylum	Class	Order	Family	Genus
T2DM	3 253 506	3 000 391	330	10	22	42	100	196
Control	6 714 732	6 244 227	403	9	22	49	117	235
Total	9 968 238	9 244 618	453	10	27	50	119	248

As the results shown in Table 3, the mean diversity of the blood microbiome (Simpson, Chao1 and Shannon indices) was not different between the T2DM cases and non‐T2DM controls at baseline. Table 4 showed the blood microbiome diversity measures by the demographic characteristics and comorbidities. No significant difference was noted relative to age, sex, smoking, drinking, or either hypertension or obesity status. In contrast, significant differences in the indices of Shannon and Simpson were observed between the participants with HDL <1.04 mmol/L and those with HDL ≥1.04 mmol/L.

**Table 3:** Richness and diversity estimators in T2DM and control groups

Indices	T2DM (n = 50)	Control (n = 100)	*P*
Chao1	2426.50 (1664.00, 2719.25)	2378.50 (1573.00, 2695.00)	0.588
Shannon	1.94 (1.74, 2.19)	1.85 (1.72, 2.21)	0.834
Simpson	0.58 (0.56, 0.61)	0.57 (0.56, 0.61)	0.984

Richness and diversity estimators were expressed as median and inter‐quartile range.

**Table 4:** Richness and diversity estimators in different groups

Chao1		Shannon		Simpson	
Age < 50 y	Age ≥ 50 y	Age < 50 y	Age ≥ 50 y	Age < 50 y	Age ≥ 50 y
2384.00 (1652.00, 2757.00)	2399.00 (1584.00, 2687.00)	1.84 (1.71, 2.18)	1.90 (1.74, 2.21)	0.57 (0.56, 0.60)	0.58 (0.56, 0.61)
Male	Female	Male	Female	Male	Female
2399.00 (1626.00, 2689.00)	2336.00 (1538.00, 2756.00)	1.85 (1.72, 2.19)	1.90 (1.74, 2.23)	0.57 (0.56, 0.61)	0.57 (0.56, 0.61)
Obese	Non‐obese	Obese	Non‐obese	Obese	Non‐obese
2049.00 (1622.50, 2570.50)	2399.00 (1593.50, 2755.50)	1.76 (1.71, 2.15)	1.94 (1.73, 2.21)	0.57 (0.56, 0.61)	0.58 (0.56, 0.61)
Diabetes	Non‐diabetes	Diabetes	Non‐diabetes	Diabetes	Non‐diabetes
2426.50 (1664.00, 2719.25)	2378.50 (1573.00, 2695.00)	1.94 (1.74, 2.19)	1.85 (1.72, 2.21)	0.58 (0.56, 0.61)	0.57 (0.56, 0.61)
Smoking	Non‐smoking	Smoking	Non‐smoking	Smoking	Non‐smoking
2408.50 (1680.75, 2689.00)	2376.00 (1532.75, 2704.50)	1.83 (1.73, 2.20)	1.93 (1.72, 2.20)	0.57 (0.56, 0.61)	0.57 (0.56, 0.61)
Drinking	Non‐drinking	Drinking	Non‐drinking	Drinking	Non‐drinking
1894.00 (1612.50, 2688.00)	2386.00 (1588.50, 2731.00)	1.75 (1.73, 2.22)	1.87 (1.72, 2.20)	0.57 (0.56, 0.61)	0.57 (0.56, 0.61)
Hypertension	Non‐hypertension	Hypertension	Non‐hypertension	Hypertension	Non‐hypertension
2249.50 (1651.50, 2709.00)	2385.00 (1590.75, 2704.50)	1.92 (1.71, 2.21)	1.86 (1.73, 2.20)	0.58 (0.56, 0.61)	0.57 (0.56, 0.61)
TC < 6.22 mmol/L	TC ≥ 6.22 mmol/L	TC < 6.22 mmol/L	TC ≥ 6.22 mmol/L	TC < 6.22 mmol/L	TC ≥ 6.22 mmol/L
2374.00 (1596.00, 2688.00)	2583.00 (1995.00, 2876.00)	1.84 (1.72, 2.20)	2.02 (1.86, 2.22)	0.57 (0.56, 0.61)	0.59 (0.57, 0.61)
TG < 2.26 mmol/L	TG ≥ 2.26 mmol/L	TG < 2.26 mmol/L	TG ≥ 2.26 mmol/L	TG < 2.26 mmol/L	TG ≥ 2.26 mmol/L
2369.00 (1580.00, 2698.00)	2434.00 (1679.50, 2726.50)	1.84 (1.74, 2.20)	1.97 (1.71, 2.20)	0.57 (0.56, 0.61)	0.58 (0.56, 0.61)
HDL < 1.04 mmol/L	HDL ≥ 1.04 mmol/L	HDL < 1.04 mmol/L	HDL ≥ 1.04 mmol/L	HDL < 1.04 mol/L	HDL ≥ 1.04 mmol/L
2112.00 (1632.50, 2580.75)	2501.00 (1587.00, 2826.50)	**1.79 (1.71, 2.12)**	**1.96 (1.74, 2.23)** *	**0.57 (0.56, 0.59)**	**0.58 (0.56, 0.61)** *
LDL < 4.14 mmol/L	LDL ≥ 4.14 mmol/L	LDL < 4.14 mmol/L	LDL ≥ 4.14 mmol/L	LDL < 4.14 mol/L	LDL ≥ 4.14 mmol/L
2384.00 (1626.00, 2689.00)	2574.00 (1550.00, 2902.00)	1.85 (1.73, 2.20)	1.97 (1.70, 2.20)	0.57 (0.56, 0.61)	0.58 (0.55, 0.61)
ALT < 40 U/L	ALT ≥ 40 U/L	ALT < 40 U/L	ALT ≥ 40 U/L	ALT < 40 U/L	ALT ≥ 40 U/L
2374.00 (1593.00, 2689.00)	2414.00 (1749.00, 2844.00)	1.84 (1.72, 2.21)	1.97 (1.75, 2.18)	0.57 (0.56, 0.61)	0.58 (0.56, 0.61)
AST < 40 U/L	AST ≥ 40 U/L	AST < 40 U/L	AST ≥ 40 U/L	AST < 40 U/L	AST ≥ 40 U/L
2386.00 (1626.00, 2707.00)	2049.00 (1468.00, 2687.00)	1.87 (1.72, 2.20)	1.80 (1.74, 2.06)	0.57 (0.56, 0.61)	0.57 (0.56, 0.59)

Richness and diversity estimators were expressed as median and inter‐quartile range.

* *P* < 0.05. The *P* values are 0.016 and 0.032 for HDL in Shannon and Simpson index analyses, respectively (in bold).

### 3.3 Associations of microbiome composition with diabetes

According to the limited criteria described in the “materials and methods,” four phyla, 14 classes, 37 orders, 97 families, and 196 genera were included for further analysis in the present study. The results showed that no significant difference in relative abundance of bacterium was detected between two groups at phylum level. Similarly, the relative abundance of bacterium in the T2DM persons was not different with the non‐T2DM controls at baseline at class level. At order level, both *Rhodospirillales* and *Myxococcales* relative levels were statistically lower in T2DM groups. At family level, the results showed that the abundances of *Burkholderiales_incertae_sedis*, *Cellulomonadaceae*, *Alteromonadaceae,* and *Chitinophagaceae* were significantly lower in cases compared to those in controls. At genus level, the *Aquabacterium, Xanthomonas, *and *Pseudonocardia* were presented with lower abundances among T2DM cases compared to those in non‐T2DM controls. However, the relative abundances of *Actinotalea*,* Alishewanella*,* Sediminibacterium*, and* Pseudoclavibacter *were much higher in T2DM cases than those in non‐T2DM controls (Table [Link], [Link], [Link], [Link], [Link], Table 5).

**Table 5:** Relative abundances of blood microbial genus in T2DM and control groups

Genus level	T2DM	Control	*P*
*Aquabacterium*	0.326 (0.047, 0.75)	0.400 (0.034, 0.785)	0.024
*Xanthomonas*	0.000 (0.000, 0.074)	0.000 (0.000, 0.237)	0.006
*Pseudonocardia*	ND	0.000 (0.000, 0.020)	0.011
*Actinotalea*	0.000 (0.000, 0.020)	0.000 (0.000, 0.013)	0.026
*Alishewanella*	0.000 (0.000, 0.014)	ND	0.045
*Sediminibacterium*	0.000 (0.000, 0.003)	0.000 (0.000, 0.003)	0.026
*Pseudoclavibacter*	0.000 (0.000, 0.007)	ND	0.014

Genus with the mean relative abundance >0.0001% was eligible for the variable selection. Relative abundances were expressed as median (maximum, minimum).

Because of low relative abundance of most pathogens in blood, we further examined the associations of blood microbiome with the onset of T2DM by characterizing the participants as carriers and non‐carriers of the pathogens. As the results shown in Table 6, individuals carried genus *Bacteroides *were significantly associated with a decreased risk of T2DM development, with 74% vs 88% (adjusted OR: 0.331, 95% CI: 0.124‐0.882, *P* = 0.0271). Participants carried the genus *Sediminibacterium *have an increased risk of T2DM, with adjusted OR (95% CI) being 14.098 (1.358, 146.330) (Table 6). However, the results from FDR correction indicated that no significant association was detected between blood microbiome and T2DM development (Table S5).

**Table 6:** Associations between blood microbiome and the subsequent risk of T2DM

Taxa	Carriers		Unadjusted		Adjusted^a^	
	T2DM (%)	Control (%)	OR (95% CI)	*P*	OR (95% CI)	*P*
*Afipia*	38	56	0.482 (0.241, 0.964)	0.0392	0.627 (0.291, 1.351)	0.2332
*Bacteroides*	74	88	0.388 (0.162, 0.930)	0.0337	0.331 (0.124, 0.882)	0.0271
*Xanthomonas*	30	53	0.380 (0.185, 0.782)	0.0086	0.500 (0.228, 1.096)	0.0835
*Actinotalea*	8	1	8.608 (0.936, 79.170)	0.0573	7.328 (0.659, 81.541)	0.1052
*Sediminibacterium*	8	1	8.608 (0.936, 79.170)	0.0573	14.098 (1.358, 146.330)	0.0267

a OR (95% CI) was adjusted by age, sex, baseline FPG, BMI, hypertension, smoking, drinking, TC, TG.

## 4 DISCUSSION

Recently, an increasing number of studies have reported that human blood contains an authentic microbiome.^18^, ^20^, ^21^, ^22^ In the present study, our data showed that the peripheral blood collected from both cases and controls has a diverse bacterial microbiota, dominated by the phyla *Proteobacteria*, *Bacteroidetes*, *Firmicutes*, and *Actinobacteria*. This result is somewhat similar to the findings reported by previous studies. The results from a France study observed that the peripheral blood from healthy donors contains bacterial DNA mostly from the *Proteobacteria* phylum (between 80.4% and 87.4%), *Actinobacteria* phylum (between 6.7% and 10.0%), the *Firmicutes* (between 3.0% and 6.4%), and *Bacteroidetes* (between 2.5% and 3.4%) phyla.^16^

To date, whether a diverse bacterial community is present in the blood of T2DM patients remains unclear. Larsen et al^23^ reported that the relative abundance of *Firmicutes* was significantly higher, whereas the proportion of *Bacteroidetes* and *Proteobacteria* were much lower in fecal in diabetic persons than their non‐diabetic counterparts. Additionally, the ratio of gut *Firmicutes* to gut *Bacteroidetes* (F/B) (the relative abundance in gut) was much higher in the patients with T2DM.^23^ However, our present data did not observe that the composition of blood microbiome in T2DM patients is different with those in non‐T2DM control at phyla level. Moreover, there was no significant difference in the value of F/B between T2DM participants and non‐T2DM controls (data not shown). It is well known that the blood microbiome is derived primarily from the gut microbiome as a result of bacterial translocation.^24^, ^25^, ^26^, ^27^ However, as previously reported, the blood and gut microbiomes differ significantly from each other, indicating that the intestinal barrier, immune cells, and liver might play a role of filtering and affecting the bacterial translocation.^28^, ^29^, ^30^, ^31^, ^32^ These results could partially explain the difference of the bacterial community diversity between the gut and blood.

In the present study, we observed that participants carried the genus *Bacteroide *were significantly associated with a decreased risk for T2DM during a 2‐year follow‐up. Previously, Yang et al^24^ observed a significant association between the presence of intestinal *Bacteroides* and obesity and diabetes. These findings indicate that the genus *Bacteroidetes* might play a protective role against T2DM development. In addition, our present results suggested that the prevalence of the genus *Sediminibacterium,* which belongs to the phylum *Proteobacteria*, was much higher in the participants who developed T2DM. However, Larsen et al^23^ have reported that the proportion of intestinal *Proteobacteria* was significantly lower in subjects with T2DM. Therefore, it is important to further investigate the mechanisms underlying the association between blood microbiome and the risk of T2DM, as it may be useful to develop microbiome‐based strategies for T2DM prevention and control.

To our knowledge, this is the first nested case‐control study to explore the role of blood microbiome in the development of T2DM. However, our present study did contain some limitations. First, we can only analyze the bacteria at the genus level in relation to T2DM risk. Whole metagenome shotgun sequencing is required for a further exploration of the microbiome at the species/strain level. Second, the information about antibiotics treatment during past six months in studied population was not collected. We cannot judge longitudinal effects of antibiotics on the blood bacterial community. Last, most of the bacterial taxa in our blood samples had a low prevalence and abundance, and our study did not have sufficient power to carry out a systematic evaluation of this. Additionally, because the results from FDR correction showed that no significant association was detected between blood microbiome and T2DM development, more investigation with a large sample size is needed in future.

## 5 CONCLUSIONS

In conclusion, our results suggested that blood microbiome may play an etiology role in the development of T2DM. In future, larger follow‐up studies are warranted in order to further determine the relationships between blood microbiome and the risk of T2DM.

## CONFLICTS OF INTEREST

The authors declare that they have no conflict of interest.

## AUTHOR CONTRIBUTIONS

JQ and YJ did the sequencing and data analyses. HZ, JQ, YJ, and CD were responsible for participants’ recruitment, consent, and sample handling. JQ and CD did the statistical analyses and data interpretation. HZ and CD wrote the manuscript, and all authors reviewed the final version.
