# Supplementary material for: Association between blood microbiome and type 2 diabetes mellitus: A nested case‐control study
Source: J Clin Lab Anal. 2019 Feb 4;33(4):e22842. doi: 10.1002/jcla.22842 (PMC6528574; doi:10.1002/jcla.22842)
Supplement: Supplementary file 1 [file JCLA-33-e22842-s001.docx]

| **Supplement table 1. Relative abundances of selected blood microbial between control and T2DM at phyla level** | | | | |
| --- | --- | --- | --- | --- |
| **Phylum** | **Control** | **DM** | **P** | **P_FDR_** |
| Firmicutes | 0.13(0.01,0.37) | 0.09(0.01,0.53) | 0.225 | 0.9 |
| Bacteroidetes | 0.15(0.01,0.42) | 0.14(0.02,0.39) | 0.603 | 1.206 |
| Actinobacteria | 0.10(0.01,0.56) | 0.11(0.03,0.44) | 0.603 | 0.804 |
| Proteobacteria | 99.55(98.77,99.96) | 99.61(98.66,99.90) | 0.862 | 0.862 |
|  |  |  |  |  |
