# Supplementary material for: Association between blood microbiome and type 2 diabetes mellitus: A nested case‐control study
Source: J Clin Lab Anal. 2019 Feb 4;33(4):e22842. doi: 10.1002/jcla.22842 (PMC6528574; doi:10.1002/jcla.22842)
Supplement: Supplementary file 2 [file JCLA-33-e22842-s002.docx]

| **Supplement table 2. Relative abundances of selected blood microbial between control and T2DM at class level** | | | | |
| --- | --- | --- | --- | --- |
| **Class** | **Control** | **T2DM** | **P** | **P_FDR_** |
| c__Sphingobacteriia | 0.00(0.00,0.05) | 0.00(0.00,0.31) | 0.108 | 1.512 |
| c__Negativicutes | 0.00(0.00,0.07) | 0.00(0.00,0.04) | 0.122 | 0.854 |
| c__Deltaproteobacteria | 0.00(0.00,0.03) | 0.00(0.00,0.03) | 0.275 | 1.283 |
| c__Epsilonproteobacteria | 0.00(0.00,0.01) | ND | 0.316 | 1.106 |
| c__Clostridia | 0.06(0.00,0.23) | 0.04(0.00,0.25) | 0.378 | 1.058 |
| c__Flavobacteriia | 0.05(0.00,0.26) | 0.04(0.00,0.16) | 0.402 | 0.938 |
| c__Gammaproteobacteria | 1.72(0.41,2.85) | 1.68(0.40,2.91) | 0.537 | 1.074 |
| c__Bacteroidia | 0.10(0.00,0.32) | 0.09(0.00,0.24) | 0.547 | 0.957 |
| c__Bacilli | 0.04(0.00,0.17) | 0.05(0.00,0.24) | 0.729 | 1.134 |
| c__Betaproteobacteria | 40.75(36.26,69.84) | 41.66(36.20,68.79) | 0.786 | 1.100 |
| c__Alphaproteobacteria | 56.91(29.70,61.11) | 56.00(30.71,61.52) | 0.839 | 1.068 |
| c__Erysipelotrichia | 0.00(0.00,0.05) | 0.00(0.00,0.02) | 0.863 | 1.007 |
| c__Actinobacteria | 0.10(0.01,0.56) | 0.11(0.03,0.44) | 0.864 | 0.930 |
| c__Cytophagia | 0.00(0.00,0.03) | 0.00(0.00,0.04) | 0.955 | 0.955 |
|  |  |  |  |  |
