# Supplementary material for: Association between blood microbiome and type 2 diabetes mellitus: A nested case‐control study
Source: J Clin Lab Anal. 2019 Feb 4;33(4):e22842. doi: 10.1002/jcla.22842 (PMC6528574; doi:10.1002/jcla.22842)
Supplement: Supplementary file 3 [file JCLA-33-e22842-s003.docx]

| **Supplement table 3. Relative abundances of selected blood microbial between control and T2DM at order level** | | | | |
| --- | --- | --- | --- | --- |
| **Order** | **control** | **T2DM** | **P** | **P_FDR_** |
| o__Rhodospirillales | 0.00(0.00,0.03) | 0.00(0.00,0.02) | 0.028 | 1.036 |
| o__Myxococcales | 0.00(0.00,0.02) | 0.00(0.00,0.01) | 0.044 | 0.814 |
| o__Legionellales | 0.00(0.00,0.01) | 0.00(0.00,0.01) | 0.1 | 1.233 |
| o__Sphingobacteriales | 0.00(0.00,0.05) | 0.00(0.00,0.31) | 0.108 | 0.999 |
| o__Selenomonadales | 0.00(0.00,0.07) | 0.00(0.00,0.04) | 0.122 | 0.903 |
| o__Caulobacterales | 0.12(0.01,1.14) | 0.09(0.01,0.22) | 0.184 | 1.135 |
| o__Alteromonadales | 0.00(0.00,0.00) | 0.00(0.00,0.01) | 0.187 | 0.988 |
| o__Chromatiales | 0.00(0.00,0.02) | 0.00(0.00,0.00) | 0.202 | 0.934 |
| o__Solirubrobacterales | 0.00(0.00,0.02) | ND | 0.216 | 0.888 |
| o__Campylobacterales | 0.00(0.00,0.01) | ND | 0.316 | 1.169 |
| o__Clostridiales | 0.06(0.00,0.23) | 0.04(0.00,0.25) | 0.365 | 1.228 |
| o__Flavobacteriales | 0.05(0.00,0.26) | 0.04(0.00,0.16) | 0.402 | 1.240 |
| o__Xanthomonadales | 0.03(0.00,0.26) | 0.03(0.00,0.10) | 0.414 | 1.178 |
| o__Aeromonadales | 0.00(0.00,0.03) | 0.00(0.00,0.02) | 0.45 | 1.189 |
| o__Gammaproteobacteria_incertae_sedis | 0.00(0.00,0.02) | ND | 0.48 | 1.184 |
| o__Pseudomonadales | 1.37(0.33,2.06) | 1.36(0.35,2.45) | 0.498 | 1.152 |
| o__Pasteurellales | 0.00(0.00,0.02) | 0.00(0.00,0.00) | 0.522 | 1.136 |
| o__Bacteroidales | 0.10(0.00,0.32) | 0.09(0.00,0.24) | 0.547 | 1.124 |
| o__Oceanospirillales | 0.00(0.00,0.03) | 0.00(0.00,0.02) | 0.57 | 1.110 |
| o__Hydrogenophilales | 0.00(0.00,0.02) | 0.00(0.00,0.02) | 0.57 | 1.055 |
| o__Bacillales | 0.03(0.00,0.14) | 0.03(0.00,0.18) | 0.573 | 1.010 |
| o__Rhizobiales | 1.80(0.24,2.65) | 1.72(0.36,3.07) | 0.582 | 0.979 |
| o__Rhodocyclales | 0.04(0.00,0.16) | 0.04(0.01,0.12) | 0.652 | 1.049 |
| o__Rhodobacterales | 0.00(0.00,0.02) | 0.00(0.00,0.01) | 0.656 | 1.011 |
| o__Methylophilales | 0.00(0.00,0.02) | 0.00(0.00,0.01) | 0.681 | 1.008 |
| o__Rubrobacterales | 0.00(0.00,0.02) | 0.00(0.00,0.04) | 0.691 | 0.983 |
| o__Neisseriales | 0.00(0.00,0.06) | 0.00(0.00,0.07) | 0.717 | 0.983 |
| o__Burkholderiales | 40.66(36.11,69.83) | 41.54(36.15,68.77) | 0.78 | 1.031 |
| o__Sphingomonadales | 53.41(29.43,58.98) | 52.83(30.19,59.58) | 0.796 | 1.016 |
| o__Bifidobacteriales | 0.00(0.00,0.03) | 0.00(0.00,0.02) | 0.835 | 1.030 |
| o__Actinomycetales | 0.10(0.01,0.55) | 0.11(0.02,0.44) | 0.839 | 1.001 |
| o__Erysipelotrichales | 0.00(0.00,0.05) | 0.00(0.00,0.02) | 0.863 | 0.998 |
| o__Bdellovibrionales | 0.00(0.00,0.02) | 0.00(0.00,0.03) | 0.863 | 0.968 |
| o__Enterobacteriales | 0.26(0.05,1.54) | 0.26(0.04,0.56) | 0.886 | 0.964 |
| o__Lactobacillales | 0.01(0.00,0.12) | 0.01(0.00,0.17) | 0.931 | 0.984 |
| o__Cytophagales | 0.00(0.00,0.03) | 0.00(0.00,0.04) | 0.955 | 0.982 |
| o__Acidimicrobiales | 0.00(0.00,0.01) | 0.00(0.00,0.01) | 0.987 | 0.987 |
|  |  |  |  |  |
