# Supplementary material for: Association between blood microbiome and type 2 diabetes mellitus: A nested case‐control study
Source: J Clin Lab Anal. 2019 Feb 4;33(4):e22842. doi: 10.1002/jcla.22842 (PMC6528574; doi:10.1002/jcla.22842)
Supplement: Supplementary file 4 [file JCLA-33-e22842-s004.docx]

| **Supplement table 4. Relative abundances of selected blood microbial between control and T2DM at family level** | | | | |
| --- | --- | --- | --- | --- |
| **Family** | **control** | **T2DM** | **P** | **P_FDR_** |
| f__Burkholderiales_incertae_sedis | 0.41(0.04,0.80) | 0.33(0.05,0.76) | 0.024 | 2.328 |
| f__Cellulomonadaceae | 0.00(0.00,0.01) | 0.00(0.00,0.02) | 0.026 | 1.261 |
| f__Chitinophagaceae | 0.00(0.00,0.00) | 0.00(0.00,0.00) | 0.026 | 0.841 |
| f__Alteromonadaceae | ND | 0.00(0.00,0.01) | 0.045 | 1.091 |
| f__Rikenellaceae | 0.00(0.00,0.03) | 0.00(0.00,0.01) | 0.052 | 1.009 |
| f__Bacillaceae 2 | 0.00(0.00,0.02) | 0.00(0.00,0.02) | 0.059 | 0.954 |
| f__Acetobacteraceae | 0.00(0.00,0.03) | 0.00(0.00,0.02) | 0.065 | 0.901 |
| f__Actinomycetaceae | 0.00(0.00,0.06) | 0.00(0.00,0.06) | 0.07 | 0.849 |
| f__Rhizobiaceae | 0.00(0.00,0.02) | 0.00(0.00,0.03) | 0.073 | 0.787 |
| f__Labilitrichaceae | 0.00(0.00,0.02) | 0.00(0.00,0.01) | 0.075 | 0.728 |
| f__Dermacoccaceae | 0.00(0.00,0.02) | 0.00(0.00,0.01) | 0.085 | 0.750 |
| f__Pseudonocardiaceae | 0.00(0.00,0.02) | 0.00(0.00,0.02) | 0.129 | 1.043 |
| f__Acidaminococcaceae | 0.00(0.00,0.01) | ND | 0.153 | 1.142 |
| f__Sphingobacteriaceae | 0.00(0.00,0.05) | 0.00(0.00,0.31) | 0.173 | 1.199 |
| f__Caulobacteraceae | 0.12(0.01,1.14) | 0.09(0.01,0.22) | 0.184 | 1.190 |
| f__Chromatiaceae | 0.00(0.00,0.02) | 0.00(0.00,0.00) | 0.202 | 1.225 |
| f__Leuconostocaceae | 0.00(0.00,0.01) | ND | 0.218 | 1.244 |
| f__Rhizobiales_incertae_sedis | 0.00(0.00,0.01) | ND | 0.218 | 1.175 |
| f__Porphyromonadaceae | 0.00(0.00,0.05) | 0.00(0.00,0.04) | 0.245 | 1.251 |
| f__Propionibacteriaceae | 0.04(0.00,0.12) | 0.04(0.00,0.28) | 0.282 | 1.368 |
| f__Brevibacteriaceae | 0.00(0.00,0.08) | 0.00(0.00,0.03) | 0.29 | 1.340 |
| f__Paenibacillaceae 1 | 0.00(0.00,0.02) | 0.00(0.00,0.05) | 0.296 | 1.305 |
| f__Thermoactinomycetaceae 1 | 0.00(0.00,0.02) | ND | 0.316 | 1.333 |
| f__Acidimicrobiaceae | 0.00(0.00,0.01) | ND | 0.316 | 1.277 |
| f__Campylobacteraceae | 0.00(0.00,0.01) | ND | 0.316 | 1.226 |
| f__Bacillaceae 1 | 0.00(0.00,0.06) | 0.00(0.00,0.07) | 0.335 | 1.250 |
| f__Veillonellaceae | 0.00(0.00,0.07) | 0.00(0.00,0.03) | 0.35 | 1.257 |
| f__Methylobacteriaceae | 0.01(0.00,0.05) | 0.01(0.00,0.07) | 0.351 | 1.216 |
| f__Halomonadaceae | 0.00(0.00,0.03) | 0.00(0.00,0.01) | 0.358 | 1.197 |
| f__Dietziaceae | 0.00(0.00,0.03) | 0.00(0.00,0.01) | 0.369 | 1.193 |
| f__Xanthomonadaceae | 0.03(0.00,0.25) | 0.03(0.00,0.10) | 0.383 | 1.198 |
| f__Lachnospiraceae | 0.02(0.00,0.12) | 0.02(0.00,0.09) | 0.397 | 1.203 |
| f__Flavobacteriaceae | 0.05(0.00,0.26) | 0.04(0.00,0.16) | 0.407 | 1.196 |
| f__Pseudomonadaceae | 0.80(0.24,1.18) | 0.77(0.25,1.29) | 0.437 | 1.247 |
| f__Hyphomicrobiaceae | 0.00(0.00,0.02) | 0.00(0.00,0.01) | 0.443 | 1.228 |
| f__Aeromonadaceae | 0.00(0.00,0.03) | 0.00(0.00,0.02) | 0.45 | 1.213 |
| f__Nocardioidaceae | 0.00(0.00,0.06) | 0.00(0.00,0.03) | 0.453 | 1.188 |
| f__Erythrobacteraceae | 0.00(0.00,0.03) | 0.00(0.00,0.01) | 0.453 | 1.156 |
| f__Geodermatophilaceae | 0.00(0.00,0.01) | 0.00(0.00,0.01) | 0.468 | 1.164 |
| f__Kineosporiaceae | 0.00(0.00,0.03) | ND | 0.48 | 1.164 |
| f__Eubacteriaceae | 0.00(0.00,0.02) | ND | 0.48 | 1.136 |
| f__Streptomycetaceae | 0.00(0.00,0.02) | ND | 0.48 | 1.109 |
| f__Aurantimonadaceae | 0.00(0.00,0.01) | 0.00(0.00,0.01) | 0.484 | 1.092 |
| f__Sutterellaceae | 0.00(0.00,0.02) | 0.00(0.00,0.01) | 0.489 | 1.078 |
| f__Ruminococcaceae | 0.02(0.00,0.09) | 0.01(0.00,0.11) | 0.492 | 1.061 |
| f__Xanthobacteraceae | 0.00(0.00,0.01) | 0.00(0.00,0.00) | 0.493 | 1.040 |
| f__Planococcaceae | 0.00(0.00,0.02) | 0.00(0.00,0.03) | 0.5 | 1.032 |
| f__Sinobacteraceae | 0.00(0.00,0.02) | 0.00(0.00,0.01) | 0.511 | 1.033 |
| f__Pasteurellaceae | 0.00(0.00,0.02) | 0.00(0.00,0.00) | 0.522 | 1.033 |
| f__Clostridiales_Incertae Sedis XI | 0.00(0.00,0.02) | 0.00(0.00,0.03) | 0.531 | 1.030 |
| f__Dermabacteraceae | 0.00(0.00,0.06) | 0.00(0.00,0.04) | 0.539 | 1.025 |
| f__Alcaligenaceae | 0.00(0.00,0.04) | 0.00(0.00,0.04) | 0.552 | 1.030 |
| f__Oxalobacteraceae | 0.03(0.00,0.48) | 0.03(0.00,0.12) | 0.558 | 1.021 |
| f__Hydrogenophilaceae | 0.00(0.00,0.02) | 0.00(0.00,0.02) | 0.57 | 1.024 |
| f__Bacillales_Incertae Sedis XII | 0.00(0.00,0.01) | 0.00(0.00,0.04) | 0.586 | 1.033 |
| f__Phyllobacteriaceae | 1.72(0.22,2.45) | 1.62(0.32,2.93) | 0.59 | 1.022 |
| f__Brucellaceae | 0.00(0.00,0.04) | 0.00(0.00,0.05) | 0.613 | 1.043 |
| f__Promicromonosporaceae | 0.00(0.00,0.03) | 0.00(0.00,0.01) | 0.623 | 1.042 |
| f__Intrasporangiaceae | 0.00(0.00,0.10) | 0.00(0.00,0.09) | 0.626 | 1.029 |
| f__Beijerinckiaceae | 0.00(0.00,0.14) | 0.00(0.00,0.04) | 0.627 | 1.014 |
| f__Bacteroidaceae | 0.02(0.00,0.11) | 0.03(0.00,0.14) | 0.63 | 1.002 |
| f__Micrococcaceae | 0.01(0.00,0.08) | 0.01(0.00,0.16) | 0.642 | 1.004 |
| f__Rhodocyclaceae | 0.04(0.00,0.16) | 0.04(0.01,0.12) | 0.652 | 1.004 |
| f__Rhodobacteraceae | 0.00(0.00,0.02) | 0.00(0.00,0.01) | 0.656 | 0.994 |
| f__Staphylococcaceae | 0.02(0.00,0.12) | 0.02(0.00,0.05) | 0.671 | 1.001 |
| f__Methylophilaceae | 0.00(0.00,0.02) | 0.00(0.00,0.01) | 0.681 | 1.001 |
| f__Rubrobacteraceae | 0.00(0.00,0.02) | 0.00(0.00,0.04) | 0.691 | 1.000 |
| f__Enterococcaceae | 0.00(0.00,0.02) | 0.00(0.00,0.01) | 0.692 | 0.987 |
| f__Aerococcaceae | 0.00(0.00,0.02) | 0.00(0.00,0.01) | 0.716 | 1.007 |
| f__Neisseriaceae | 0.00(0.00,0.06) | 0.00(0.00,0.07) | 0.717 | 0.994 |
| f__Bacteriovoracaceae | 0.00(0.00,0.02) | 0.00(0.00,0.03) | 0.727 | 0.993 |
| f__Tsukamurellaceae | 0.00(0.00,0.02) | 0.00(0.00,0.00) | 0.75 | 1.010 |
| f__Nocardiaceae | 0.00(0.00,0.02) | 0.00(0.00,0.03) | 0.753 | 1.001 |
| f__Burkholderiaceae | 0.10(0.01,0.26) | 0.09(0.01,0.31) | 0.756 | 0.991 |
| f__Mycobacteriaceae | 0.00(0.00,0.02) | 0.00(0.00,0.03) | 0.759 | 0.982 |
| f__Comamonadaceae | 38.74(34.85,69.07) | 39.44(35.29,67.98) | 0.762 | 0.973 |
| f__Nocardiopsaceae | 0.00(0.00,0.01) | 0.00(0.00,0.01) | 0.777 | 0.979 |
| f__Prevotellaceae | 0.05(0.00,0.21) | 0.05(0.00,0.15) | 0.78 | 0.970 |
| f__Clostridiaceae 1 | 0.00(0.00,0.01) | 0.00(0.00,0.02) | 0.785 | 0.964 |
| f__Bradyrhizobiaceae | 0.02(0.00,0.08) | 0.02(0.00,0.06) | 0.786 | 0.953 |
| f__Sphingomonadaceae | 53.34(29.42,58.92) | 52.78(30.19,59.56) | 0.799 | 0.957 |
| f__Carnobacteriaceae | 0.00(0.00,0.02) | 0.00(0.00,0.01) | 0.805 | 0.952 |
| f__Corynebacteriaceae | 0.01(0.00,0.22) | 0.01(0.00,0.06) | 0.814 | 0.951 |
| f__Rhodospirillaceae | 0.00(0.00,0.03) | 0.00(0.00,0.02) | 0.814 | 0.940 |
| f__Bifidobacteriaceae | 0.00(0.00,0.03) | 0.00(0.00,0.02) | 0.835 | 0.953 |
| f__Alicyclobacillaceae | 0.00(0.00,0.02) | 0.00(0.00,0.02) | 0.851 | 0.960 |
| f__Erysipelotrichaceae | 0.00(0.00,0.05) | 0.00(0.00,0.02) | 0.863 | 0.962 |
| f__Enterobacteriaceae | 0.26(0.05,1.54) | 0.26(0.04,0.56) | 0.886 | 0.977 |
| f__Moraxellaceae | 0.54(0.07,1.63) | 0.54(0.09,1.16) | 0.892 | 0.972 |
| f__Lactobacillaceae | 0.00(0.00,0.02) | 0.00(0.00,0.01) | 0.895 | 0.965 |
| f__Microbacteriaceae | 0.00(0.00,0.18) | 0.00(0.00,0.05) | 0.906 | 0.966 |
| f__Bdellovibrionaceae | 0.00(0.00,0.02) | 0.00(0.00,0.01) | 0.961 | 1.013 |
| f__Streptococcaceae | 0.01(0.00,0.07) | 0.01(0.00,0.07) | 0.966 | 1.008 |
| f__Cytophagaceae | 0.00(0.00,0.03) | 0.00(0.00,0.04) | 0.971 | 1.002 |
| f__Nakamurellaceae | 0.00(0.00,0.02) | 0.00(0.00,0.02) | 0.987 | 1.008 |
| f__Bacillales_Incertae Sedis XI | 0.00(0.00,0.01) | 0.00(0.00,0.01) | 0.992 | 1.002 |
| f__Peptostreptococcaceae | 0.00(0.00,0.04) | 0.00(0.00,0.01) | 1 | 1.000 |
|  |  |  |  |  |
