# Supplementary material for: Association between blood microbiome and type 2 diabetes mellitus: A nested case‐control study
Source: J Clin Lab Anal. 2019 Feb 4;33(4):e22842. doi: 10.1002/jcla.22842 (PMC6528574; doi:10.1002/jcla.22842)
Supplement: Supplementary file 5 [file JCLA-33-e22842-s005.docx]

| **Supplement table 5. Relative abundances of selected blood microbial between control and T2DM at genus level** | | | |  |
| --- | --- | --- | --- | --- |
| **Genus** | **control** | **T2DM** | **P** | **P_FDR_** |
| g__Xanthomonas | 0(0,0.237) | 0(0,0.074) | 0.006 | 1.176 |
| g__Pseudonocardia | 0(0,0.02) | ND | 0.011 | 1.078 |
| g__Pseudoclavibacter | ND | 0(0,0.007) | 0.014 | 0.915 |
| g__Aquabacterium | 0.4(0.034,0.785) | 0.326(0.047,0.75) | 0.024 | 1.176 |
| g__Actinotalea | 0(0,0.013) | 0(0,0.02) | 0.026 | 1.019 |
| g__Sediminibacterium | 0.000(0.000,0.003) | 0.000(0.000,0.003) | 0.026 | 0.849 |
| g__Alishewanella | ND | 0(0,0.014) | 0.045 | 1.260 |
| g__Alistipes | 0(0,0.03) | 0(0,0.014) | 0.052 | 1.274 |
| g__Brevibacillus | 0(0,0.014) | 0(0,0.054) | 0.069 | 1.503 |
| g__Finegoldia | 0(0,0.01) | 0(0,0.014) | 0.069 | 1.352 |
| g__Labilithrix | 0(0,0.017) | 0(0,0.014) | 0.075 | 1.336 |
| g__Yimella | 0(0,0.02) | ND | 0.078 | 1.274 |
| g__Cupriavidus | 0(0,0.014) | 0(0,0.007) | 0.081 | 1.221 |
| g__Afipia | 0(0,0.034) | 0(0,0.02) | 0.085 | 1.190 |
| g__Parabacteroides | 0(0,0.051) | 0(0,0.044) | 0.093 | 1.215 |
| g__Clostridium XlVa | 0(0,0.027) | 0(0,0.017) | 0.097 | 1.188 |
| g__Zhihengliuella | 0(0,0.017) | 0(0,0.01) | 0.1 | 1.153 |
| g__Vogesella | 0(0,0.01) | 0(0,0.01) | 0.1 | 1.089 |
| g__Actinomyces | 0(0,0.057) | 0(0,0.057) | 0.114 | 1.176 |
| g__Anoxybacillus | 0(0,0.051) | 0(0,0.044) | 0.12 | 1.176 |
| g__Tepidimonas | 0(0,0.03) | 0(0,0.017) | 0.135 | 1.260 |
| g__Gordonia | 0(0,0.014) | 0(0,0.012) | 0.138 | 1.229 |
| g__Marmoricola | 0(0,0.017) | 0(0,0.003) | 0.14 | 1.193 |
| g__Sphingobacterium | 0(0,0.034) | 0(0,0.314) | 0.142 | 1.160 |
| g__Limnohabitans | 0.01(0,0.027) | 0.01(0,0.02) | 0.143 | 1.121 |
| g__Acidovorax | 0.06(0,0.142) | 0.074(0.007,0.192) | 0.144 | 1.086 |
| g__Devosia | 0(0,0.017) | 0(0,0.01) | 0.149 | 1.082 |
| g__Moraxella | 0(0,0.049) | ND | 0.153 | 1.071 |
| g__Paenibacillus | 0(0,0.017) | ND | 0.153 | 1.034 |
| g__Phascolarctobacterium | 0(0,0.013) | ND | 0.153 | 1.000 |
| g__Butyricimonas | 0(0,0.01) | ND | 0.153 | 0.967 |
| g__Cytophaga | 0(0,0.01) | ND | 0.153 | 0.937 |
| g__Brevundimonas | 0.1(0.007,1.109) | 0.086(0.014,0.196) | 0.156 | 0.927 |
| g__Nesterenkonia | ND | 0(0,0.02) | 0.157 | 0.905 |
| g__Azoarcus | ND | 0(0,0.02) | 0.157 | 0.879 |
| g__Chryseobacterium | 0.01(0,0.203) | 0.01(0,0.078) | 0.189 | 1.029 |
| g__Lysinibacillus | 0(0,0.003) | 0(0,0.003) | 0.195 | 1.033 |
| g__Rheinheimera | 0(0,0.02) | 0(0,0.003) | 0.202 | 1.042 |
| g__Roseomonas | 0(0,0.03) | 0(0,0.007) | 0.204 | 1.025 |
| g__Psychrobacter | 0(0,0.007) | ND | 0.208 | 1.019 |
| g__Skermanella | 0(0,0.003) | 0(0,0.017) | 0.212 | 1.013 |
| g__Azospirillum | 0(0,0.03) | ND | 0.218 | 1.017 |
| g__Sutterella | 0(0,0.02) | ND | 0.218 | 0.994 |
| g__Leuconostoc | 0(0,0.013) | ND | 0.218 | 0.971 |
| g__Varibaculum | 0(0,0.01) | ND | 0.218 | 0.950 |
| g__Nitratireductor | 0(0,0.007) | 0(0,0.01) | 0.218 | 0.929 |
| g__Geodermatophilus | 0(0,0.01) | ND | 0.218 | 0.909 |
| g__Proteiniclasticum | 0(0,0.003) | 0(0,0.01) | 0.218 | 0.890 |
| g__Porphyromonas | 0(0,0.03) | 0(0,0.024) | 0.224 | 0.896 |
| g__Aeromicrobium | 0(0,0.044) | 0(0,0.03) | 0.224 | 0.878 |
| g__Megamonas | 0(0,0.024) | 0(0,0.02) | 0.242 | 0.930 |
| g__Porphyrobacter | 0(0,0.027) | 0(0,0.014) | 0.244 | 0.920 |
| g__Trabulsiella | 0(0,0.01) | 0(0,0.003) | 0.261 | 0.965 |
| g__Ruminococcus | 0(0,0.024) | 0(0,0.017) | 0.263 | 0.955 |
| g__Propionibacterium | 0.04(0,0.122) | 0.042(0.003,0.277) | 0.266 | 0.948 |
| g__Rhizobium | 0(0,0.024) | 0(0,0.03) | 0.271 | 0.949 |
| g__Pedobacter | 0(0,0.02) | 0(0,0.01) | 0.282 | 0.970 |
| g__Granulicatella | 0(0,0.007) | 0(0,0.007) | 0.287 | 0.970 |
| g__Brevibacterium | 0(0,0.078) | 0(0,0.027) | 0.29 | 0.963 |
| g__Paraprevotella | 0(0,0.027) | 0(0,0.027) | 0.29 | 0.947 |
| g__Gemmiger | 0(0,0.03) | 0(0,0.024) | 0.302 | 0.970 |
| g__Thermoactinomyces | 0(0,0.02) | ND | 0.316 | 0.999 |
| g__Clostridium XVIII | 0(0,0.014) | ND | 0.316 | 0.983 |
| g__Rubellimicrobium | 0(0,0.014) | ND | 0.316 | 0.968 |
| g__Vasilyevaea | 0(0,0.014) | ND | 0.316 | 0.953 |
| g__Curtobacterium | 0(0,0.014) | ND | 0.316 | 0.938 |
| g__Selenomonas | 0(0,0.01) | ND | 0.316 | 0.924 |
| g__Salmonella | 0.02(0,0.247) | 0.022(0,0.138) | 0.317 | 0.914 |
| g__Geobacillus | 0(0,0.02) | 0(0,0.027) | 0.323 | 0.918 |
| g__Schlegelella | 0.03(0,0.129) | 0.03(0,0.118) | 0.328 | 0.918 |
| g__Leucobacter | 0(0,0.024) | 0(0,0.017) | 0.329 | 0.908 |
| g__Methylobacterium | 0.01(0,0.051) | 0.012(0,0.067) | 0.355 | 0.966 |
| g__Massilia | 0.02(0,0.297) | 0.02(0,0.095) | 0.36 | 0.967 |
| g__Halomonas | 0(0,0.024) | 0(0,0.01) | 0.363 | 0.961 |
| g__Nocardiopsis | 0(0,0.01) | 0(0,0.003) | 0.363 | 0.949 |
| g__Blautia | 0(0,0.027) | 0(0,0.014) | 0.364 | 0.939 |
| g__Kytococcus | 0(0,0.017) | 0(0,0.007) | 0.366 | 0.932 |
| g__Dietzia | 0(0,0.028) | 0(0,0.007) | 0.369 | 0.927 |
| g__Amaricoccus | 0(0,0.017) | 0(0,0.007) | 0.375 | 0.930 |
| g__Clostridium XI | 0(0,0.02) | 0(0,0.003) | 0.375 | 0.919 |
| g__Jeotgalicoccus | 0(0,0.054) | 0(0,0.01) | 0.382 | 0.924 |
| g__Adhaeribacter | 0(0,0.01) | 0(0,0.003) | 0.388 | 0.927 |
| g__Shinella | 0(0,0.02) | 0(0,0.007) | 0.443 | 1.046 |
| g__Aeromonas | 0(0,0.034) | 0(0,0.02) | 0.45 | 1.050 |
| g__Bacillus | 0(0,0.03) | 0(0,0.041) | 0.451 | 1.040 |
| g__Novosphingobium | 0.07(0.01,0.186) | 0.081(0.01,0.182) | 0.471 | 1.073 |
| g__Hymenobacter | 0(0,0.014) | 0(0,0.01) | 0.475 | 1.070 |
| g__Kingella | 0(0,0.037) | ND | 0.48 | 1.069 |
| g__Kineosporia | 0(0,0.027) | ND | 0.48 | 1.057 |
| g__Sporosarcina | 0(0,0.02) | ND | 0.48 | 1.045 |
| g__Fibrella | 0(0,0.02) | ND | 0.48 | 1.034 |
| g__Mitsuokella | 0(0,0.017) | ND | 0.48 | 1.023 |
| g__Eubacterium | 0(0,0.017) | ND | 0.48 | 1.012 |
| g__Streptomyces | 0(0,0.017) | ND | 0.48 | 1.001 |
| g__Butyricicoccus | 0(0,0.014) | 0(0,0.012) | 0.484 | 0.999 |
| g__Veillonella | 0(0,0.027) | 0(0,0.017) | 0.485 | 0.990 |
| g__Atopostipes | 0(0,0.014) | 0(0,0.014) | 0.486 | 0.982 |
| g__Xanthobacter | 0(0,0.01) | 0(0,0.003) | 0.493 | 0.986 |
| g__Microbacterium | 0(0,0.041) | 0(0,0.014) | 0.496 | 0.982 |
| g__Bosea | 0(0,0.017) | 0(0,0.02) | 0.496 | 0.972 |
| g__Caulobacter | 0.01(0,0.057) | 0.007(0,0.051) | 0.497 | 0.964 |
| g__Sphingopyxis | 0(0,0.017) | 0(0,0.017) | 0.499 | 0.959 |
| g__Faecalibacterium | 0.01(0,0.074) | 0.007(0,0.044) | 0.505 | 0.961 |
| g__Pseudomonas | 0.77(0.239,1.178) | 0.752(0.25,1.263) | 0.506 | 0.954 |
| g__Coprococcus | 0(0,0.024) | 0(0,0.034) | 0.507 | 0.946 |
| g__Nevskia | 0(0,0.017) | 0(0,0.007) | 0.511 | 0.945 |
| g__Escherichia/Shigella | 0.19(0.024,1.383) | 0.176(0.017,0.458) | 0.522 | 0.956 |
| g__Sandaracinobacter | 0(0,0.007) | 0(0,0.003) | 0.527 | 0.956 |
| g__Serinicoccus | 0(0,0.014) | 0(0,0.01) | 0.53 | 0.953 |
| g__Azonexus | 0(0,0.014) | 0(0,0.007) | 0.537 | 0.957 |
| g__Solibacillus | 0(0,0.02) | 0(0,0.017) | 0.539 | 0.952 |
| g__Enhydrobacter | 0(0,0.068) | 0.003(0,0.02) | 0.558 | 0.977 |
| g__Dialister | 0(0,0.024) | 0(0,0.01) | 0.562 | 0.975 |
| g__Delftia | 0.52(0.034,1.022) | 0.517(0.122,0.935) | 0.563 | 0.968 |
| g__Phyllobacterium | 1.71(0.219,2.449) | 1.616(0.324,2.927) | 0.569 | 0.970 |
| g__Bdellovibrio | 0(0,0.02) | 0(0,0.007) | 0.58 | 0.980 |
| g__Bacteriovorax | 0(0,0.014) | 0(0,0.027) | 0.585 | 0.980 |
| g__Exiguobacterium | 0(0,0.01) | 0(0,0.037) | 0.586 | 0.973 |
| g__Brachybacterium | 0(0,0.058) | 0(0,0.037) | 0.591 | 0.973 |
| g__Lachnospiracea_incertae_sedis | 0(0,0.027) | 0(0,0.017) | 0.601 | 0.982 |
| g__Zoogloea | 0(0,0.01) | 0(0,0.01) | 0.609 | 0.986 |
| g__Odoribacter | 0(0,0.024) | 0(0,0.008) | 0.616 | 0.990 |
| g__Flectobacillus | 0(0,0.01) | 0(0,0.014) | 0.618 | 0.985 |
| g__Solobacterium | 0(0,0.027) | 0(0,0.007) | 0.623 | 0.985 |
| g__Novispirillum | 0(0,0.017) | 0(0,0.003) | 0.623 | 0.977 |
| g__Aureimonas | 0(0,0.013) | 0(0,0.003) | 0.623 | 0.969 |
| g__Beijerinckia | 0(0,0.142) | 0(0,0.037) | 0.627 | 0.968 |
| g__Capnocytophaga | 0(0,0.02) | 0(0,0.01) | 0.628 | 0.962 |
| g__Bacteroides | 0.02(0,0.108) | 0.027(0,0.135) | 0.63 | 0.957 |
| g__Pelomonas | 0(0,0.017) | 0(0,0.226) | 0.639 | 0.963 |
| g__Anaerococcus | 0(0,0.02) | 0(0,0.034) | 0.642 | 0.961 |
| g__Staphylococcus | 0.02(0,0.118) | 0.017(0,0.054) | 0.645 | 0.958 |
| g__Rothia | 0(0,0.024) | 0(0,0.047) | 0.661 | 0.974 |
| g__Paracoccus | 0(0,0.007) | 0(0,0.01) | 0.664 | 0.971 |
| g__Serpens | 0(0,0.007) | 0(0,0.003) | 0.671 | 0.974 |
| g__Bradyrhizobium | 0.01(0,0.071) | 0.01(0,0.041) | 0.674 | 0.971 |
| g__Arthrobacter | 0(0,0.02) | 0(0,0.014) | 0.679 | 0.971 |
| g__Dechloromonas | 0.04(0,0.152) | 0.042(0.003,0.115) | 0.687 | 0.976 |
| g__Leadbetterella | 0(0,0.017) | 0(0,0.017) | 0.687 | 0.969 |
| g__Rhodococcus | 0(0,0.024) | 0(0,0.017) | 0.688 | 0.963 |
| g__Rubrobacter | 0(0,0.024) | 0(0,0.044) | 0.691 | 0.961 |
| g__Barnesiella | 0(0,0.013) | 0(0,0.03) | 0.691 | 0.954 |
| g__Enterococcus | 0(0,0.024) | 0(0,0.01) | 0.692 | 0.948 |
| g__Ochrobactrum | 0(0,0.03) | 0(0,0.01) | 0.702 | 0.956 |
| g__Xylophilus | 0(0,0.013) | 0.003(0,0.01) | 0.704 | 0.952 |
| g__Pseudorhodoferax | 0(0,0.01) | 0(0,0.003) | 0.705 | 0.946 |
| g__Methylotenera | 0(0,0.007) | 0(0,0.003) | 0.705 | 0.940 |
| g__Herbaspirillum | 0(0,0.024) | 0(0,0.017) | 0.717 | 0.950 |
| g__Ralstonia | 0.09(0.007,0.254) | 0.081(0.01,0.304) | 0.72 | 0.947 |
| g__Ruminococcus2 | 0(0,0.02) | 0(0,0.007) | 0.722 | 0.943 |
| g__Peptoniphilus | 0(0,0.014) | 0(0,0.02) | 0.724 | 0.940 |
| g__Blastomonas | 0.06(0.003,0.159) | 0.054(0.007,0.121) | 0.726 | 0.936 |
| g__Clostridium sensu stricto | 0(0,0.01) | 0(0,0.02) | 0.729 | 0.934 |
| g__Neisseria | 0(0,0.034) | 0(0,0.071) | 0.747 | 0.951 |
| g__Salinicoccus | 0(0,0.014) | 0(0,0.013) | 0.749 | 0.947 |
| g__Tsukamurella | 0(0,0.017) | 0(0,0.003) | 0.75 | 0.942 |
| g__Mycobacterium | 0(0,0.02) | 0(0,0.034) | 0.759 | 0.948 |
| g__Flavobacterium | 0.02(0,0.115) | 0.017(0,0.071) | 0.76 | 0.943 |
| g__Nocardioides | 0(0,0.02) | 0(0,0.017) | 0.76 | 0.937 |
| g__Altererythrobacter | 0(0,0.01) | 0(0,0.01) | 0.763 | 0.935 |
| g__Sphingomonas | 51.604(29.046,57.715) | 51.146(29.759,58.297) | 0.768 | 0.935 |
| g__Kocuria | 0(0,0.064) | 0(0,0.037) | 0.787 | 0.952 |
| g__Duganella | 0(0,0.02) | 0(0,0.007) | 0.789 | 0.949 |
| g__Comamonas | 0.69(0.088,1.581) | 0.714(0.145,1.374) | 0.792 | 0.947 |
| g__Pseudochrobactrum | 0(0,0.02) | 0(0,0.027) | 0.796 | 0.946 |
| g__Acinetobacter | 0.44(0.067,1.014) | 0.472(0.064,1.134) | 0.802 | 0.947 |
| g__Pedomicrobium | 0(0,0.01) | 0(0,0.014) | 0.806 | 0.946 |
| g__Variovorax | 36.860(32.917,68.619) | 37.385(33.675,67.367) | 0.814 | 0.950 |
| g__Bifidobacterium | 0(0,0.027) | 0(0,0.014) | 0.82 | 0.951 |
| g__Fusicatenibacter | 0(0,0.017) | 0(0,0.014) | 0.821 | 0.947 |
| g__Clostridium XlVb | 0(0,0.02) | 0(0,0.01) | 0.825 | 0.946 |
| g__Sphingobium | 0.43(0.044,0.74) | 0.429(0.057,0.77) | 0.826 | 0.941 |
| g__Alloprevotella | 0(0,0.024) | 0(0,0.017) | 0.828 | 0.938 |
| g__Cloacibacterium | 0(0,0.074) | 0(0,0.024) | 0.834 | 0.939 |
| g__Hydrogenophilus | 0(0,0.02) | 0(0,0.02) | 0.834 | 0.934 |
| g__Micrococcus | 0(0,0.051) | 0(0,0.074) | 0.851 | 0.948 |
| g__Alicyclobacillus | 0(0,0.024) | 0(0,0.017) | 0.851 | 0.942 |
| g__Prevotella | 0.05(0,0.203) | 0.044(0,0.154) | 0.859 | 0.946 |
| g__Corynebacterium | 0.01(0,0.112) | 0.012(0,0.064) | 0.864 | 0.946 |
| g__Cellvibrio | 0.01(0,0.057) | 0.01(0,0.044) | 0.89 | 0.969 |
| g__Lactobacillus | 0(0,0.02) | 0(0,0.01) | 0.895 | 0.969 |
| g__Roseburia | 0(0,0.017) | 0(0,0.014) | 0.918 | 0.989 |
| g__Streptococcus | 0.01(0,0.068) | 0.008(0,0.068) | 0.927 | 0.993 |
| g__Wautersiella | 0(0,0.03) | 0(0,0.027) | 0.945 | 1.007 |
| g__Burkholderia | 0(0,0.064) | 0(0,0.078) | 0.946 | 1.002 |
| g__Parasutterella | 0(0,0.01) | 0(0,0.01) | 0.946 | 0.997 |
| g__Janibacter | 0(0,0.094) | 0(0,0.084) | 0.959 | 1.005 |
| g__Dorea | 0(0,0.01) | 0(0,0.014) | 0.959 | 1.000 |
| g__Methylobacillus | 0(0,0.017) | 0(0,0.014) | 0.981 | 1.017 |
| g__Peredibacter | 0(0,0.024) | 0(0,0.013) | 0.985 | 1.016 |
| g__Alcaligenes | 0(0,0.034) | 0(0,0.034) | 0.987 | 1.013 |
| g__Facklamia | 0(0,0.024) | 0(0,0.007) | 0.987 | 1.008 |
| g__Kurthia | 0(0,0.007) | 0(0,0.034) | 0.987 | 1.002 |
| g__Megasphaera | 0(0,0.017) | 0(0,0.02) | 0.987 | 0.997 |
| g__Nakamurella | 0(0,0.017) | 0(0,0.017) | 0.987 | 0.992 |
| g__Gemella | 0(0,0.013) | 0(0,0.014) | 0.992 | 0.992 |
|  |  |  |  |  |
